# Assessing the Pharmacological and Therapeutic Efficacy of Traditional Chinese Medicine Liangxue Tongyu Prescription for Intracerebral Hemorrhagic Stroke in Neurological Disease Models

**DOI:** 10.3389/fphar.2018.01169

**Published:** 2018-11-06

**Authors:** Xun Li, Xi Huang, Yuanlin Tang, Fangli Zhao, Yanmei Cao, Lian Yin, Guochun Li

**Affiliations:** ^1^College of Pharmacy, Nanjing University of Chinese Medicine, Nanjing, China; ^2^Jiangsu Wujin Vocational School, Changzhou, China; ^3^College of Preclinical Medical, Nanjing University of Chinese Medicine, Nanjing, China

**Keywords:** intracerebral hemorrhage, liangxue tongyu prescription, network pharmacology, compound combination, PI3K/AKT

## Abstract

Intracerebral hemorrhage is a fatal subtype of stroke, with crucial impact on public health. Surgical removal of the hematoma as an early-stage treatment for ICH can’t improve long-term prognosis remarkably. Liangxue tongyu prescription (LP), a Traditional Chinese Medicine (TCM) formula, includes eight ingredients and has been used to treat ICH in the clinical. In the study, we elucidated the pharmacological efficacy and therapeutic efficacy of LP to dissect the mechanism of LP against ICH via network analysis and experimental validation. First, we discovered 34 potential compounds and 146 corresponding targets in LP based on network prediction. 24 signal pathway were obtained by the Clue Go assay based on potential compounds in LP against ICH. Second, we found that LP can not only decreased the level of high sensitive C reactive protein (HS-CRP), tumor necrosis factor-α (TNF-α), NF-kβ, D-dimmer (D2D), estradiol (E2), S-100B, neuron specific enolase (NSE), and interleukin 1 (IL-1) in plasma on spontaneously hypertensive rats (SHRs), but also promoted cell proliferation and inhibited cell apoptosis on the glutamate-induced PC12 cell. The compounds including Taurine, Paeonol, and Ginsenoside Rb1 in LP can activate PI3K/AKT pathway. Third, from the three-factor two-level factorial design, compound combinations in LP, such as Taurine and Paeonol, Taurine and Geniposide, Ginsenoside Rg1, and Ginsenoside Rb1, had first-level interactions on cell proliferation. Compound combinations including Taurine and Paeonol, Ginsenoside Rg1 and Ginsenoside Rb1 had as significant increase in efficiency on inhibiting the apoptosis of PC12 cells at the low concentration and up-regulating of PI3K and AKT. Overall, our results suggested that LP had integrated therapeutic effect on ICH due to activities of anti-inflammatory, anti-coagulation, blood vessel protection, and protection neuron from excitotoxicity based on the way of “multi-component, multi-target, multi-pathway,” and compound combination in LP can offer protection neuron from excitotoxicity at the low concentration by activation of the PI3K/Akt signal pathway.

## Introduction

Intracerebral hemorrhagic (ICH) stroke is one of devastating diseases accompanied with high percentages of morbidity and mortality, causing approximately 15% of deaths from all strokes (Feigin et al., 2009; van Asch et al., 2010; Steiner et al., 2014). Brain injury following ICH is a dynamic process involving a cascade of complex pathological pathways and biochemical and metabolic events, which is approximately categorized as hemorrhagic neuroinflammation, decrease of blood flow, cytotoxic and vasogenic edema, free radical injury, rise of intracranial pressure, neuronal apoptosis and brain herniation (Lok et al., 2011; Zhou et al., 2014). These events cause recurrent strokes to make more damage to brain. However, surgical removal of the hematoma can’t improve long-term prognosis dramatically, and no effective targeted therapy for hemorrhagic stroke exists yet.

Traditional Chinese Medicine (TCM) has always played a crucial role in maintaining health for over thousands of years. Indeed, prescription is the most common clinical practice of TCM, which combines several types of botanical drugs or animal medicines or minerals to fight with various diseases. Liangxue tongyu prescription (LP) is a TCM formula against stroke diseases in the clinic (Ma et al., 2011; Shi et al., 2017) including eight ingredients, namely, eight ingredients, namel *Paeonia lactiflora* Pall. (PR), *Rheum* *officinale* Baill. (RO), *Rehmannia glutinosa* (Gaertn.) Li-bosch. (RG), *Panax notoginseng* (Burk.) F. H. Chen ex C. Chow. (PN), *Paeonia suffruticosa* Andr. (PS), *Acorus tatarinowii* Schott. (AT), *Bubalus bubalis* Linnaeus. (BB), *Pheretima aspergillum* (E. Perrier) (PA). Based on classical TCM theory, LP was created by Chen et al. (2016), a leading expert in the field of traditional Chinese medicine. Clinical data have proved that LP exerted its comprehensive therapeutic effects on ICH through ameliorating permeability of blood brain barrier, reducing secondary brain edema, promoting hacmatoma absorption, alleviating neuroinflammation and neurological damage (Zhang et al., 2012; Huang et al., 2014). According to the theory of TCM, LP exerts its healing effects on ICH with the effect of cooling-blood and activating-blood. Volatile oil and *n*-butanol extracts in LP were primarily identified as active fractions of LP with the effect of cooling-blood and activating-blood (Huang X. et al., 2015). However, the pharmacological effects and therapeutic efficacy of LP against ICH remain a mystery.

**FIGURE 1:**
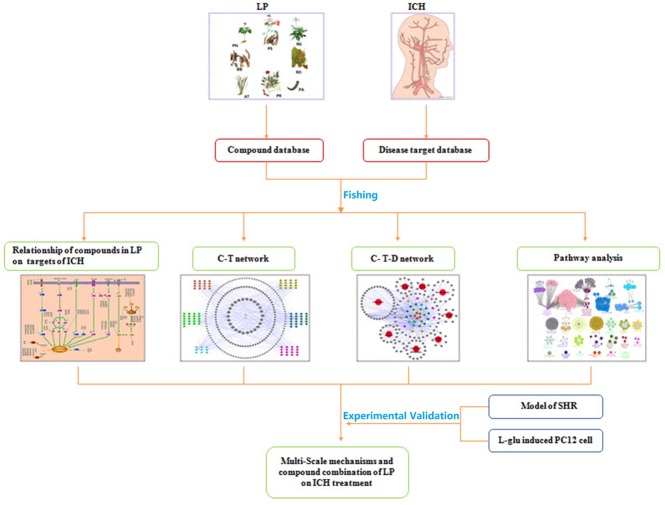
Network pharmacology approach framework.

Based on the characteristics of complex compounds in LP, unclear targets and systemic therapy, a workflow for network-based TCM approach was proposed on ICH. The overall procedure is illustrated in Figure 1. It starts with the identification of compounds present in LP and corresponding targets on ICH, and ends with analyzing the signaling pathways and sub-networks regulated by compounds of LP and evaluating its effects on ICH network. Further, the network prediction results were verified *in vitro* and *in vivo* experiments. On one hand, the model of spontaneously hypertensive rat (SHR) and *L*-glutamate-damaged PC12 cells were administered to investigate the pharmacological effects of LP on ICH. On the other hand, compound combination in LP were induced to verify therapeutic efficacy of LP on the *L*-glutamate-damaged PC12 cells by the PI3K-AKT pathway.

## Materials and Methods

### ICH Associated Genes

Multiple genes of ICH were gathered in the GeneCards^1^ (a database about all known diseases with a genetic component and references for further research and tools for genomic analysis of a cataloged gene), Online Mendelian Inheritance in Man database (OMIM, a database about genes, their products and biomedical applications, maintained by Israel’s Weizmann Institute of Science) and related literatures with keywords of ‘hemorrhagic stroke’ and ‘intracranial hemorrhage.’ Finally, 1436 distinct targets associated with ICH diseases were dug out.

### All Compounds in LP

Compounds of each ingredient in LP were collected from TCM Systems Pharmacology Database^2^ (TcmSP^TM^, a unique systems pharmacology platform designed for herbal medicines) and pertinent literatures. Totally, 574 compounds were collected in LP. Separately, there were 120 compounds in PR, 93 compounds in RO, 77 compounds in RG, 56 compounds in PS, 120 compounds in PN, and 106 compounds in AT. However, almost no distinctive compound was found from ingredient BB except for taurine and PA except for lumbrokinase.

### The Properties of Compounds in LP

Six significant pharmacology-related properties including number of acceptor atoms for H-bonds (nHAcc), molecular weight (MW), number of donor atoms for H-bonds (nHDon), Moriguchi octanol–water partition coeff (logP) (MLOGP), oral bioavailability (OB), and drug-likeness (DL) were acquired from TcmSP^TM^, which reflected fundamental characteristics of each ingredient (Lipinski et al., 1997). The properties of each ingredient were analyzed based on the physicochemical properties of all compounds with *t*-test.

### Predicting Target Profiles of Compounds in LP

All targets profiles associated with compounds in LP were collected from SciFinder and PubMed. Partial targets involve gene and protein files of different species, so we limited the entering species to “Homo sapiens” in the NCBI dabase^3^ by putting in each target that does not meet racial requirements, standardization of targets files could be got by revising to their official names. Though the transformation and retrieval, exclusive target files related with compounds in LP were finally obtained.

### Oral Bioavailability and Drug-Likeness Screening

The OB value was calculated with the in-house system OBioavail 1.1, which was applied to select out the potential compounds. In this paper, those compounds with OB≧30% were chosen as potential compounds for subsequent study, and DL value of a new compound was figured by Tanimoto similarity.

$$\text{f(a, b)=}\frac{\text{a}\cdot \text{b}}{|a{|}^{2}+|b{|}^{2}-\text{a}\cdot \text{b}}$$

where ‘*a*’ represents the new compound, and ‘*b*’ is the average molecular properties of all compounds in Drug-Bank database (Xu et al., 2012). Through the method, compounds which satisfied the requirements with OB≧30% and DL≧0.1 were chosen as potential compounds.

### Systems Pharmacology Network Construction and Analysis

(1) Compound-target network (C-T network). The compounds of LP and corresponding ICH targets were applied to create C-T network with the network visualization software Cytoscape 2.8.2 (Shannon et al., 2003). (2) Potential compound-target-disease network (P-T-D network). Potential compounds of LP, targets and diseases were employed to build the P-T-D network using network visualization software Cytoscape 2.8.2. (3) Pathway analysis. 142 potential corresponding targets of potential compounds in LP were carried out pathway analysis with GlueGo to analyze biological interpretation and interrelations of functional targets in biological networks.

### Experimental Validation

#### Materials

*Paeonia lactiflora* Pall. (20140901), *Rheum* *officinale* Baill. (20140601), *Rehmannia glutinosa*(Gaertn.)Li-bosch. (20140102), *Panax notoginseng* (Burk.) F. H. Chen ex C. Chow. (20151021), *Paeonia suffruticosa* Andr. (20140901), *Acorus tatarinowii* Schott. (20140301), *Bubalus bubalis* Linnaeus (20140601), *Pheretima aspergillum* (E. Perrier). (20140601), were purchased from Tong Leng Hetian Chinese medicine company.

#### Extraction Preparation

Total extract of LP (TLP): Heat reflux extraction with water technique was applied to obtain TLP. At first, all ingredients of LP were dry matter and smashed. Then 1 kg mixture of LP was soaked in distilled water for 60 min, extracted in a 20 L round-bottomed flask containing 10 L of distilled water for 1 h and repeated heat reflux extraction for 1 h with10 L of new distilled water again. Then double extraction solution were combined and evaporated under reduced pressure to remove most solvents by using a rotary evaporator. Finally the extraction was dried into lyophilized powder. A total of lyophilized powder of 641.15 g was got with 5 Kg mixtures of LP.

*n*-Butanol fraction extract (LPB): A total of 5 Kg mixture of LP was extracted with heat reflux extraction with water, Then double extraction solution were combined, evaporated and extracted by equivalent volume of *n*-butanol at five times. Finally, the *n*-butanol fraction liquid was combined, evaporated and dried into lyophilized powder of 109.60 g.

Volatile oil fraction extract (LPV): 1 Kg mixture of LP was put in a 20 L round-bottomed flask containing 10 L of distilled water, which was connected to a Clevenger-type apparatus with tap water for cooling. The mixture of LP was under method of heat reflux extraction with water for 1 h. Meanwhile, the obtained essential oil was collected in the side arm, then separated and dried with anhydrous sodium sulfate to eliminate moisture. Finally, the volatile oil of 9.10 ml was obtained with 5 Kg mixtures of LP (á = 0.91 g/ml).

#### Durgs and Reagents

Aspirin was purchased from by Bayer HealthCare with the batch number of J20130078). Enzyme-linked Immunosorbent Assay (ELISA) Kits including Rat interleukin 1 (IL-1), Rat S-100B protein, Rat nuclear factorκB (NF-κB), Rat neuron specific enolase (NSE), Rat high sensitive C reactive protein (HS-CRP), Rat estradiol (E2), Rat tumor necrosis factor-α (TNF-α), and Rat D-dimmer (D2D), were purchased from Nanjing Senbeijia Biological Technology Co., Ltd., Trizol was purchased from Invitrogen. Anti-AKT, Anti-PI3K, and Anti-β-actin antibody was all purchased from Abcam. All durgs (purity assay by HPLC ≥ 98%, power) including compounds of Emodin, Ginsenoside Rg1, Ginsenoside Rb1, Notoginsenoside R1, Baicalein, Geniposide, β-asarone, Taurine, Paeoniflorin, Paeonol, Apigenin, Rhein, and Catalpol, were purchased from Chengdu Must Bio-Technology Co., Ltd.

#### Animals and Prescription Administration

48-male-(8-week-old) SHR (180–210 g), 8 male Wistar Rats (180–210 g) were purchased from Vital River Laboratory Animal Technology Co. Ltd. (License: No. SCXK (Jing) 2012-0001). All mice were housed with standard controlled conditions (12/12 h light/dark with humidity of 40–60%, 21 ± 2°C), and were allowed free available to food and water. Then they were acclimatized in the laboratory environment for a week prior to the experiment. The health condition of rat was checked regularly.

Eight Wistar rats as blank control groups (Distilled Water group), 48 SHR were at random assigned into six groups (*n* = 8 in each group) including model group (SHR group only), positive control group (Aspirin), LPB high dosage group, LPB low dosage group, LPV high dosage group, and LPV low dosage group. Administration of gastric infusion was executed in the dose of 10 mL/kg once a day in each group, 15 days in total. On the 15th day, 1 h after administration, draw 5 ml blood from carotid artery, and transfer it into centrifuge tube containing 0.038 g/ml sodium citrate. Centrifuge at 3000 rpm for 15 min. Collect the top layer of plasma to determine indexes of NSE, IL-1, S-100B, NF-κB, TNF-α, E2, D2D, and HS-CRP.

#### Cells Culture and Treatment

PC12 cells, kindly donated from Professor Lu (Department of Chinese Medicine Pharmacology, Nanjing University of Chinese Medicine) were cultured at 37°C in DMEM containing 10% (v/v) heat-inactivated fetal bovine serum (FBS; GIBCO), 100 U/ml penicillin and 100 μg/ml streptomycin (Hyclone, J150019) under a humidified atmosphere of 95% air and 5% CO_2_. For cell differentiation, cells were treated with 50 ng/mL of nerve growth factor (NGF; Sigma-Aldrich, USA) for 48 h. Afterwards, NGF-differentiated cultures were pretreated with different doses of each medicine at 1, 5, 10, and 20 μmol/L for 1 h, and then expored to10 mM *L*-glutamate for an additional 24 h. The untreated PC cells without the treatment of *L*-glutamate were used as control group.

#### Cell Counting Kit-8 Assay

PC12 cells were seeded into 96-well plates at a density of 50 cells/μL and were treated by drugs in the same way as described above, and then gently washed with PBS. Afterwards, 10 μl of CCK-8 solution and 90 μl of culture medium were added to each well. The 96-well plates were maintained at 37°C for 4 h and the absorbance was measured in a microplate reader (Rayto; RT-6000) at 450 nm. All groups were repeated in triplicate.

#### Flow Cytometry (FCM) With Propidium Iodide (PI) Staining

The PC12 cells were seeded in 6-well plates at a density of 1 × 10^5^/ml and incubated for 24 h. Cells were treated by drugs in the same way as described above. Cells were treated with10 mM glutamate for 24 h, then colleted and harvested with 0.25% trypsin and made into a single cell suspension, washed with cold Phosphate buffer (PBS) twice. PC12 Cells were resuspended as a single cell suspension with 50 μl PBS, fixed with l ml of cold 70% ethanol. Then 5 μl of Annexin V-FITC and 5 μl of PI stained cells in order. The cells were incubated at room temperature (20–25°C)for 15 min in the dark and determined with a FACS Calibur flow cytometer (Becton–Dickinson).

#### Real-Time Quantitative Polymerase Chain Reaction

PC12 cells were seeded in 6 well plates and then resuspended at 1200 rpm for 10 min in DMEM. Total RNA was extracted with Trizol reagent (Invitrogen, America), and the concentration of total RNA was measured with Merinton SMA4000 and the purity was estimated with the ratio of A260/A280 between 2.0 and 2.3. The first strand of cDNA was obtained with Prime Script^TM^ RT Master Mix (Takara), and expression levels of mRNA was quantified with real-time PCR amplification instrument (Eppendorf, Germany). The program was included 1 cycle of 95°C for 5 min, 40 cycles of 95°C for 5 s, 60°C for 31 s. The mRNA levels of each gene took mRNA of GAPDH as the standard. The specific primers used in the reaction were as follows. The experiment was conducted in triplicate.

**Table:** 

Gene	Forward primer	Reverse primer
PI3K	GATGATTTACGGCAAGATA	CACCACCTCAATAAGTCCC
AKT	CGGCAAGGTGATCCTGGTG	CGGTCGTGGGTCTGG≧G
GAPDH	CTGGGCTACACTGAGCACC	AAGTGGTCGTTGAGGGCAATG

#### Western Blotting

The PC12 cells were washed with pre-cooled PBS buffer for 3 times, and lysed with RIPA buffer (100 μl/50 ml) (Beyotime, Shanghai, China) and then placed for 30 min on ice. Cells were harvested by scraping, centrifuged at 12,000 rpm for 10 min. Bovine serum albumin (BSA) (2 μg/μl) was diluted in PBS, and protein concentration was detected with BCA Protein Assay Kit (Pierce, Thermo Scientific) at a 50:1 ratio. The lysates (2 μl) were diluted in double-distilled water (18 μl) (each sample in two wells). 200 μl of detection solution were added to each well in a 96-well plates. The OD values of samples were tested at a wave length of 490, and standard curve was drawn to calculate the concentrations of the proteins for every sample. The samples were first electrophoresed at 60 V, and then the voltage was rised to 120 V as soon as the samples reached the separation gel, followed by electrophoresis (conducted at 4°C) for 2 h. After electrophoresis, the proteins were transferred onto polyvinylidene fluoride (PVDF) membranes for 2 h (conducted at 4°C). The PVDF was removed from the transfer apparatus, and the samples were blocked with 5% evaporated milk in tris-buffered salinetween (TBST) and incubated at room temperature for 1 h. The membranes were individually incubated with anti-phospho-Akt(1:1,000, Abcam, United Kingdom) and anti-phospho-PI3K (1:1,000, Abcam, United Kingdom) with the samples at 4°C overnight. After three washes with TPBS, secondary antibodies (HRP; l:5000, Abcam, United Kingdom) corresponded to the respective primary antibodies at room temperature for 1 h. The images were captured with the Clinx3200 + System (Clinx Science Instruments Co., Ltd., Shanghai, China), and protein densitometry was analyzed with Quantity One software (Bio-Rad, Hercules, CA, United States). Each membrane was stripped and quantified as a ratio to β-actin.

### Statistical Analysis

Comparisons among multiple groups were analyzed by one-way analysis of variance (ANOVA) with *post hoc* test (Bonferroni or Dunnett’s correction for multiple tests). For comparison between two groups, normal distribution data was analyzed by two-tailed unpaired Student’s *t*-test and non-normally distributed data was analyzed by Kruskal–Wallis tests. Statistical analysis was carried out using SPSS18.0 software. *P* < 0.05 or *P* < 0.01 was considered as statistically significant.

## Results

### Excavation of Compounds Database of LP and Targets Database on ICH

One thousand, four hundred and thirty-six distinct targets associated with ICH were collected from the OMIM and GenesCard database, which basically included the important disease genes of cerebral hemorrhage. Among the 1436 target genes of ICH, inflammatory response associated genes took the lion’s share with 681 targets which were 47.42% of the total disease target genes. In addition, apoptosis associated genes had577 associated genes, accouting for 40.18% of ICH targets.

A total of 572 compounds of six botanical herbs in LP were acquired from TCMSP^TM^, Drug Bank, TTD, and related literatures, from which 120 compounds in PR, 93 in RO, 77 in RG, 56 in PS, 120 in PN, and 106 in AT were all collected. In addition, PA and BB have abundant amino acids from recent literatures (Liu et al., 2015; Yu et al., 2017).

### Network Analysis of Compounds in LP on ICH

**FIGURE 2:**
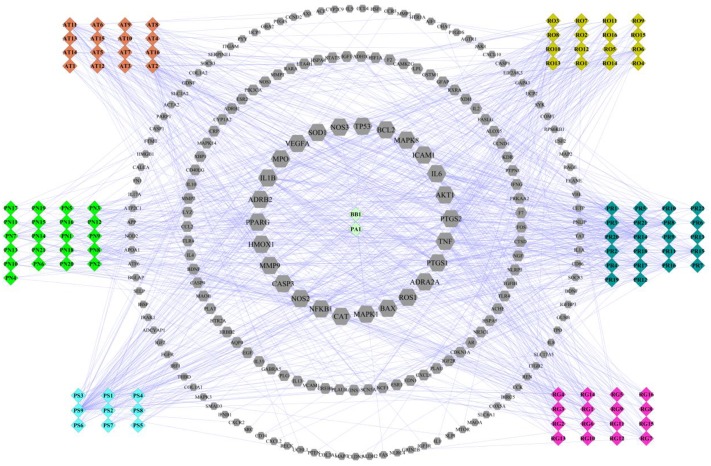
Compound-target network of liangxue tongyu prescription (LP) on intracerebral hemorrhage. The figure was constructed by combining 102 compounds and 200 corresponding targets which were validated in published literature. Diamond represents compounds. Every color represents the single medicine. Gray hexagon represents target. Targets in the internal circle with bigger size display more relationships with compounds than these in the outside circles. PR, *Paeonia lactiflora* Pall.; RO, *Rheum* *officinale* Baill.; RG, *Rehmannia glutinosa* (Gaertn.) Li-bosch.; PN, *Panax notoginseng* (Burk.) F. H. Chen ex C. Chow.; PS, *Paeonia suffruticosa* Andr.; AT, *Acorus tatarinowii* Schott.; BB, *Bubalus bubalis* Linnaeus.; PA, *Pheretima aspergillum* (E. Perrier).

For deep understanding of the complex relationship of compounds in LP and targets of ICH from the molecular level, visual network of compounds and their corresponding targets (C-T network) was constructed. At the outset, compared compounds in LP and targets of ICH, those genes that could be targeted by the corresponding compound were selected out. Then 102 compounds in LP and 200 corresponding targets were found. Second, C-T network was created with network visualization software Cytoscape. the C-T network contains 302 nodes which represents targets and compounds, and 1018 compound-target interactions (Figure 2). The mean degree value of compounds which is defined as the amount of target related to it was 9.92, indicating that most compounds exerted comprehensive effects by regulating multiple targets. Meanwhile, the external circle of targets showed lower degree value and the innermost circle of targets had the highest degree value with 22.923. It indicated that these targets may have more important positions for LP against ICH. For instance, activation of matrix metalloproteinase-9 (MMP9) can compromise blood–brain barrier, and aggravate proinflammatory gene expression and neutrophils infiltration during the formation of e perihematomal edema (Min et al., 2015). Tumor Protein P53 (TP53) gene affected mobilization of endogenous marrow-derived EPC to promote neovascularization and improve functional prognosis after ICH (Rodriguez et al., 2017). Therefore, these targets of higher value may become important therapeutic aims of LP.

**FIGURE 3:**
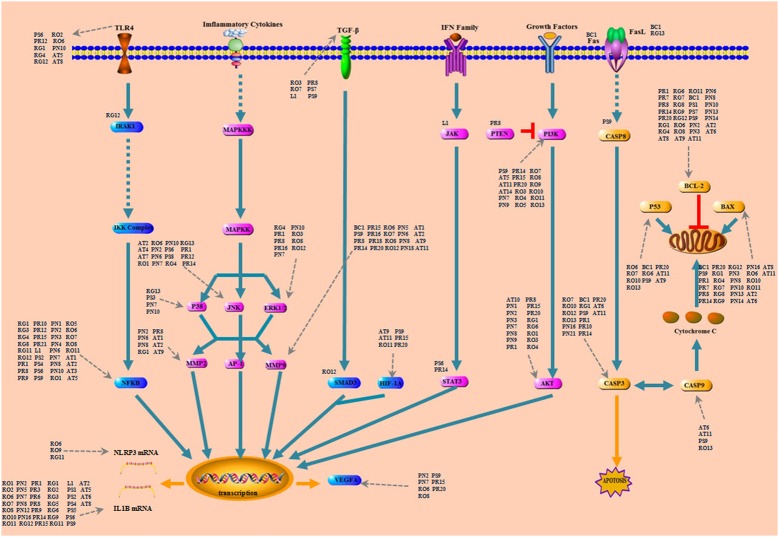
Compounds and hitting targets on ICH. It showed an intuitional three-level structure that multi-compound hit groups of targets through multi-pathway to play the regulative role. All pathways were involved in compound control process. The nodes are pathway protein targets and the surrounding represent compounds of LP. PR, *Paeonia lactiflora* Pall.; RO, *Rheum* *officinale* Baill.; RG, *Rehmannia glutinosa* (Gaertn.) Li-bosch.; PN, *Panax notoginseng* (Burk.) F. H. Chen ex C. Chow.; PS, *Paeonia suffruticosa* Andr.; AT, *Acorus tatarinowii* Schott.; BB, *Bubalus bubalis* Linnaeus.; PA, *Pheretima aspergillum* (E. Perrier).

Intracerebral hemorrhage is a dynamic process involving a cascade of complex pathological pathways. LP performed treating effects with its active compounds binding and regulating particular protein or nucleic acid targets. Based on the C-T network, the relationship of compounds in LP on targets of ICH was further analyzed. As shown in Figure 3, abundant compounds in LP regulated signaling pathways by adjusting and controlling these key pathway targets on ICH. Some important common pathways such as TGF-β/smad signaling, Mitogen-Activated Protein Kinase Cascades, Apoptosis Cascades,PI3K/AKT signaling, Toll-like Receptors Pathway and NF-κB Signaling were included in the course of ICH process (Liu et al., 2016; Taylor et al., 2017; Wen et al., 2017), which covered apoptosis, proliferation, differentiation, inflammatory and immunoregulation and many other important biological processes. Meanwhile, a total of 72 compounds from eight ingredients of LP affected the prothrombotic and proinflammatory signaling pathways and regulated the AKT, MAPK, and BCL-2 pathways. These compounds may regulate the same target with coordination and antagonism or independent relationship. In conclusion, LP exerted its effects on ICH with multiple compounds regulating multiple pathways.

### Network and Pathway Analysis of Potential Compounds in LP on ICH

#### Potential Compounds in LP Screening

With the purpose of screening out the potential compounds in LP, OB and DL evaluation were applied. According to screening rules of OB≧30% and DL≧0.1, a total of 32 potential compounds of six herbal medicines in LP were selected out (Table 1). However, some compounds among them which couldn’t meet the requirements, were confirmed by experimental evidence and then were also allocated to potential compounds. For example, Catalpol has been displayed that it had the properties of anti-apoptosis, anti-inflammation and neuroprotection against Alzheimer’s disease, ischemic stroke and idiopathic Parkinson’s disease (Jiang et al., 2015). Geniposide enhanced growth factor signaling and inhibited apoptosis of neurone in the mouse model of Parkinson disease to exert neuroprotective effect (Jiang et al., 2015). β-Asarone was also added as potential compound for further analysis because β-Asarone treatment could suppress Beclin-1-dependent autophagy through the PI3K/Akt/mTOR signal pathway (Deng et al., 2016). *Panax* *notoginseng* saponins (PNS) were mainly composed of Ginsenoside Rb1, Ginsenoside Rg1, and Notoginsenoside R1,which had positive protection on ischemia brain damage with the activity of *anti-inflammation* and anti-apoptosis (Huang X.P. et al., 2015). Paeonol may possess anti-inflammatory and anti-oxidant functions with its active metabolites (Jin et al., 2016). In addition, Taurine was found that it promoted synapse development and proliferation of neuron (Liu et al., 2010). Lumbrokinase may relieve ischemia-reperfusion injury via TLR4 signaling (Wang et al., 2016). In all, It was found that 34 potential compounds in LP were preserved for subsequent analysis.

**Table 1:** The potential compounds in herbology of Liangxuet Tongyu Prescription (LP).

NO	Compound	OB	DL	NO	Compound	OB	DL
PR1	Paeoniflorin	53.87	0.79	RG12	β-Sitosterol	36.91	0.75
PR3	Ethyl linoleate	42	0.19	RG14	Stigmasterol	43.87	0.76
PR4	Albiflorin	30.25	0.77	PS5	Hederagenin	36.91	0.75
PR8	Paeonol	28.79	0.04	PS7	Betulinic acid	55.38	0.78
PR10	Catechin	54.83	0.24	PS9	Quercetin	46.43	0.28
PR14	Kaempferol	41.88	0.24	PN1	Ginsenosides Rg1	-	-
PR15	Naringenin	33.23	0.24	PN2	Ginsenoside Rb1	6.24	0.04
PR19	Oleic acid	33.13	0.14	PN3	Notoginsenoside R1	5.43	0.13
PR20	Baicalein	33.52	0.21	PN17	(-)-alpha-Cedrene	55.56	0.10
RO5	Piceatannol	72.29	0.13	PN19	Diop	43.59	0.39
RO7	Emodin	24.4	0.24	PN20	Mandenol	42	0.19
RO8	Rhein	47.07	0.28	AT2	β-Asarone	35.61	0.06
RO10	Aloe-emodin	83.38	0.24	AT5	Astragalin	14.03	0.74
RO14	Eupatin	50.8	0.41	AT7	N-*trans*-feruloyltyramine	86.71	0.26
RG1	Catalpol	5.07	0.44	AT11	Apigenin	23.06	0.21
RG6	Geniposide	3.78	0.44	AT14	Marmesin	50.28	0.18

*PR, Paeonia lactiflora Pall.; RO, Rheum officinale Baill.; RG, Rehmannia glutinosa (Gaertn.) Li-bosch.; PN, Panax notoginseng (Burk.) F. H. Chen ex C. Chow.; PS, Paeonia suffruticosa Andr.; AT, Acorus tatarinowii Schott. “—”represents uncollected information*.

#### Network Analysis of Potential Compounds in LTF on ICH

**FIGURE 4:**
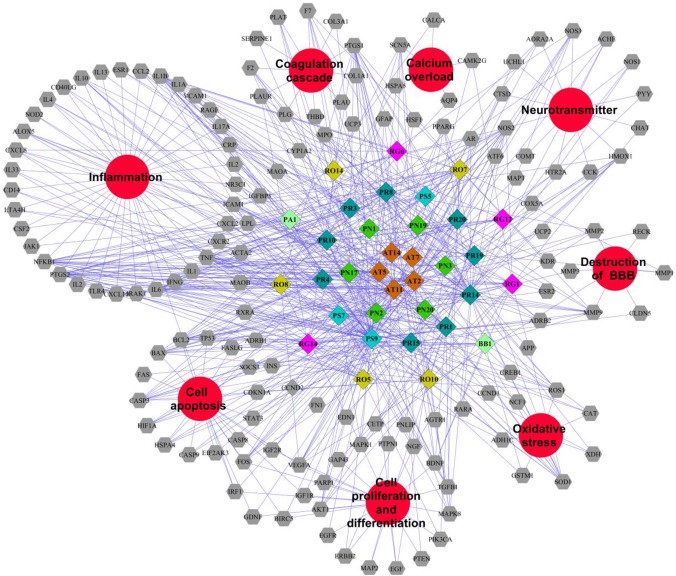
Potential compound-targets-disease network in LP on ICH. There were 34 potential compounds, 146 corresponding targets and eight pathologyies types on ICH in the P-T-D network. Diamond represents potential compounds, each color represents one herb in LP respectively; Gray hexagon represents targets while the red circles represent the pathologyies types of ICH. PR, *Paeonia lactiflora* Pall.; RO, *Rheum* *officinale* Baill.; RG, *Rehmannia glutinosa* (Gaertn.) Li-bosch.; PN, *Panax notoginseng* (Burk.) F. H. Chen ex C. Chow.; PS, *Paeonia suffruticosa* Andr.; AT, *Acorus tatarinowii* Schott.; BB, *Bubalus bubalis* Linnaeus.; PA, *Pheretima aspergillum* (E. Perrier).

Traditional Chinese Medicine which involved many bioactive compounds may hit one or multiple targets from the network of biology. Then the biological system achieved new balance to reduce the harmful effects. Typically, Potential compound-targets- disease (P-T-D) network in LP on ICH was constructed to forecast and interpret the poly pharmacology action of multi-compound and multi-target. As shown in Figure 4, there are 34 potential compounds and 146 corresponding targets with the connections also reaching 506 items which indicates the close relationships between them. Lots of associated targets independently belong to inflammation (36/146), cell apoptosis (21/146), cell proliferation and differentiation (22/146), oxidative stress (7/146), destruction of blood-brain barrier (7/146), neurotransmitter (13/146), calcium overload(5/146), and coagulation cascade (11/146), which implied that the compounds of LP may intervene many pathologies of ICH such as neurologic function, thrombosis, oxidative stress, cell apoptosis, and inflammation. For example, prothrombin (F2) and coagulation factor VII (F7) are important coagulation factors in the thrombosis process. The injured vessels induce these circulating coagulation factors in a chain of interlacing reactions, causing development of a coagulum to generate thrombosis, and multi-compound in LP might regulate coagulation factors to put into effect.

#### Pathway Analysis of Targets Associated With Potential Compounds in LP on ICH

**FIGURE 5:**
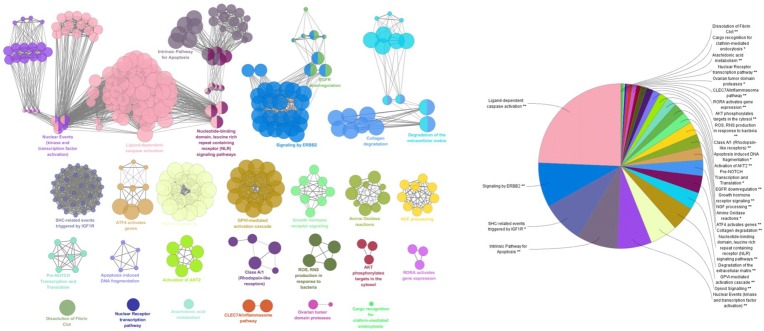
ClueGO founctional analysis of the potential targets. GO terms and pathways representative for target genes are selected and visualized with a functionally grouped network, the GO terms render as nodes, and the significance is represented with node size. Functionally related groups are able to partially overlap. The node pie charts stand for the reactome analysis of targets. Only the most important term was labeled. The above GlueGO setting was representative reactome analysis among potential targets.

A total of 142 corresponding targets rooted in P-T-D network were chosen as potential ICH targets to carry on Go analysis. As shown in Figure 5, 24 signaling pathways of ICH associated with potential compounds in LP were fully excavated by REACTOME pathways with 9841 available unique genes. The main pathways were involved in inflammation, coagulation, apoptosis and other pathologic links and relevant signaling pathways. Specifically, the reactome pathways were mainly related to Ligand-dependent caspase activation, signaling by ERBB2, SHC-related events triggered by IGF1R and intrinsic pathway for Apoptosis. Moreover, although some pathways didn’t occupy larger proportion, they also display tremendous importance, such as dissolution of Fibrin Clot, inflammasome pathway, AKT phosphorylates targets in the cytosol, and degradation of the extracellular matrix. These reactome pathways have been linked to the internal mechanism of hemorrhagic stroke and indicated the complexity of disease pathological process in more depth. Protein targets of ICH were shown to be involved in autophagy, ischemia, necrosis, apoptosis, calpain activation, inflammation, oxidative stress, caspase activation and cytokine secretion based on the current level of global pathway analysis. Go analysis resulted that these potential protein targets of potential compounds in LP were involved in specific pathological pathways basically.

**FIGURE 6:**
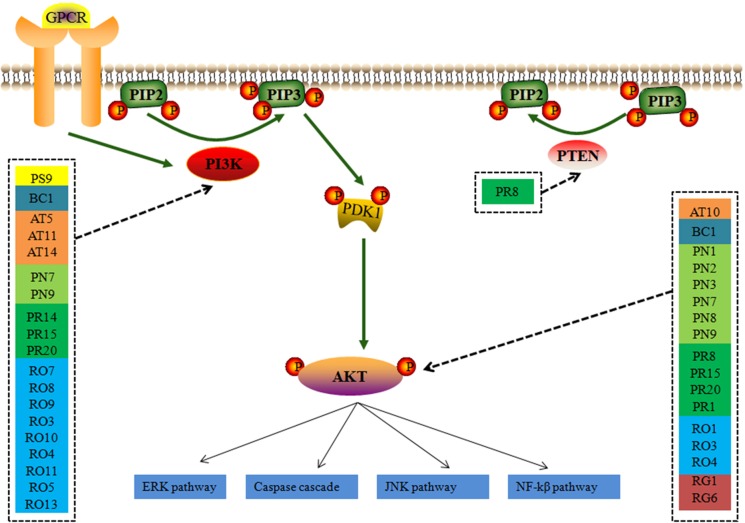
The simplified PI3K-AKT pathway regulation of compounds in LP. Radix *Paeoniae* Rubra. RO, *Rheum* *officinale* Baill.; RG, *Rehmannia glutinosa* (Gaertn.) Li-bosch.; PN, *Panax notoginseng* (Burk.) F. H. Chen.; PS, *Paeonia suffruticosa* Andr.; AT, *Acorus tatarinowii* Schott.; BB, *Bubalus bubalis* Linnaeus.; PA, *Pheretima aspergillum* (E. Perrier).

The perihematomal region resulted in a prominent form of cell death during the development of ICH. Therefore, inhibition of the neuron apoptosis has been expected to improve functional recovery and reduce tissue damage in ICH. Among the pathways of ICH associated with potential compounds in LP, the PI3K-AKT pathway wasn’t dominant position, but it had obvious upstream and downstream proteins which played a significant role in neuronal apoptosis, and an important contributor to early brain injury after ICH by taking part in cell survival and proliferation and regulating some important caspase proteins and apoptotic genes. As shown in Figure 6, PI3K stimulated the generation of the lipid second messenger, converting phosphatidylinositol (4,5) bisphosphate (PIP2) into phosphatidylinositol (3,4,5) trisphosphate (PIP3). PTEN antagonized action of PI3K, and Akt resorted its pleckstrin homology domain to bind the 3^′^-phosphorylated inositol lipids. Compounds in LP have interferential effect on the phosphorylation of PI3K and AKT proteins based on the dissection of network pharmacology. PI3K may be targeted by 19 compounds from eight ingredients of LP such as Emodin, Baicalein, Ginsenoside Rg1, and so on. AKT may be targeted by 17 compounds from eight ingredients of LP such as Catalpol, Paeoniflorin, Paeonol, Ginsenoside Rg1, Ginsenoside Rb1, and so on. PI3K inhibitor could be targeted by only one compound Paeonol. The PI3K-Akt pathway acted an important part in inhibition of apoptosis and neuroprotection (Wang et al., 2013). The downsteam target AKT influences many other pathways such as inhibition of caspase and the regulation of Bcl-2 family proteins, NF-kβ pathway, JNK pathway, and ERK pathway (Jacquin et al., 2013; McNamara et al., 2013; Zhou et al., 2015; Velmurugan et al., 2017).

### Experimental Verification on ICH

#### Pharmacodynamic Evaluation of LP on SHR Model

Based on the network pharmacological prediction, the compounds of LP may act on different pathologies of ICH such as neurologic function, thrombosis and inflammation. ICH stands for the acute presentation of cerebral small-vessel disease. Persistently raised intraluminal arterial pressure destories small-vessel walls and hypertension becomes the leading cause of ICH (Biffi et al., 2015). The level of IL-1, HS-CRP, NF-κB, S-100B protein, TNF-α, NSE, D2D, and E2 were detected in SHR treated with extracted fractions from LP. The LPV and LPB, two effective extracted fractions in LP for anti-inflammatory and activating blood on blood-heat and blood-stasis rat model, were adopted to estimate pharmacodynamic evaluation of LP on SHR model.

**FIGURE 7:**
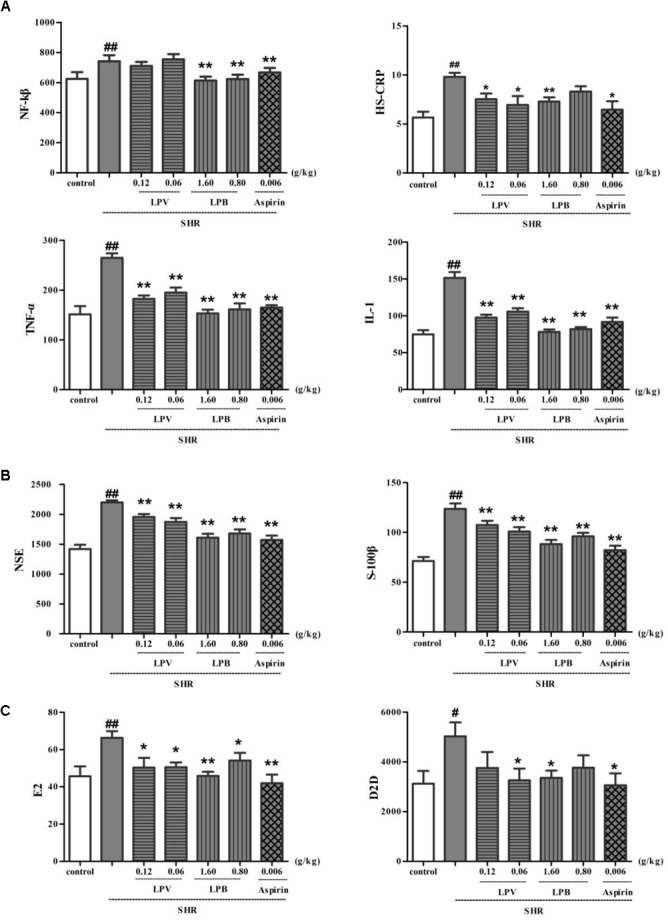
Effects of LPB and LPV from LP and aspirin on levels of IL-1, NF-κB, TNF-α, HS-CRP **(A)**, NSE, S-100B **(B)**, D2D and E2 **(C)** in serum on SHR model. Data were expressed as the mean ± SEM for six rat (^∗^*P* < 0.05, ^∗∗^*P* < 0.01, vs. SHR group only (model group); ^#^*P* < 0.05, ^##^*P* < 0.01, vs. Control). LP, Liangxue tongyu prescription; LPV, Volatile oil fraction extract of LP; LPB, *n*-butanol fraction extracts of LP; Glu, glutamate; SHR, spontaneously hypertensive rat.

High sensitive C reactive protein, TNF-α, NF-kβ, and IL-1 levels in plasma in model group were obviously higher than that in control group (*P* < 0.01, Figure 7A). HS-CRP, TNF-α, NF-kβ, and IL-1 levels in aspirin group as positive medicine (Chong et al., 2012) was observably lower than that in model group (*P* < 0.01, *P* < 0.05). Compared to model group, after treatment with different doses of LPB and LPV, HS-CRP, TNF-α and IL-1 levels in serum were all significantly decreased (*P* < 0.05) and NF-kβ levels in LPB treatment groups were all significantly decreased (*P* < 0.01).

S-100B and NSE level in model groups increased remarkably in contrast to control group (*P* < 0.01, Figure 7B). In comparison with model group, level of S-100B and NSE in aspirin, LPV and LPB groups decreased significantly (*P* < 0.01). Compared with control group, D2D and E2 level in model group show a significant difference (*P* < 0.01, Figure 7C). Compared with model group, D2D and E2 level of aspirin, LPV and LPB groups reduced significantly (*P* < 0.05, *P* < 0.01). The D2D is very sensitive to intravascular thrombus and may be markedly elevated in disseminated intravascular coagulation. E2 exerted a protective effect by intracellular Ca^2+^ levels (Koh, 2014).

#### Effects of Extracted Fractions and Compounds of LP on *L*-Glutamate-Induced PC12 Cell Proliferation

Cell proliferation was evaluated on the glutamate-induced PC12 cell treated with extracted fractions and compounds of LP. As is shown in Table 2, *L*-glutamate obviously inhibited cell proliferation in comparison to control group (*p* < 0.01), different dosages of LPB and TLP all can enhanced proliferation of PC12 cells (*p* < 0.01). The group dealt with LBV only enhanced cell viability at 6.435 μg/mL (*p* < 0.01).

**Table 2:** Effects of extract of LP and its extracted fractions including LPB, LPV, and TLP on the cell proliferation in L-Glu-damaged PC12 cell.

Group	Dose (μg/mL)	OD450
Control	-	1.292 ± 0.081
Glu	-	0.891 ± 0.054ˆ##
Glu + TLP	64.118	1.166 ± 0.120ˆ**
Glu + TLP	128.235	1.208 ± 0.057ˆ**
Glu + TLP	256.469	1.300 ± 0.063ˆ**
Glu + TLP	512.938	1.302 ± 0.052ˆ**
Glu + LPB	10.959	1.640 ± 0.037ˆ**
Glu + LPB	21.917	1.208 ± 0.043ˆ**
Glu + LPB	43.833	1.307 ± 0.038ˆ**
Glu + LPB	87.665	1.333 ± 0.065ˆ**
Glu + LPV	0.818	0.944 ± 0.012
Glu + LPV	1.636	0.967 ± 0.021
Glu + LPV	3.723	1.018 ± 0.023
Glu + LPV	6.545	1.125 ± 0.051ˆ**

*Data are expressed with Mean ± SEM. ^#^P < 0.05, ^##^P < 0.01, vs. control. ^∗^P < 0.05, ^∗∗^P < 0.01, vs. model (only Glu-damaged group). Each extract has four doses corresponding to the dose of crude medicine at 4000, 2000, 1000, and 500 μg/ml. Glu:glutamate, LPV, Volatile oil fraction extract of LP; LPB, n-butanol fraction extracts of LP; TLP, total extract of LP; LP, liangxue tongyu prescription*.

**FIGURE 8:**
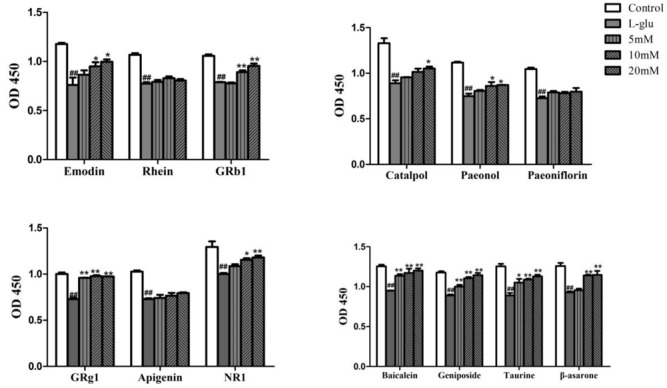
Effects of compounds in LP on the cell proliferation on L-Glu-damaged PC12 cell. Data are expressed as mean ± SEM. ^#^P < 0.05, ^##^P < 0.01, vs. control. ^∗^P < 0.05, ^∗∗^P < 0.01, vs. model (only Glu-damaged group). Glu, glutamate; GRg1, ginsenoside rg1; GRb1, ginsenoside rb1; NR1, Notoginsenoside R1.

In the meanwhile, Emodin, GRb1, Catalpol, Paeonol, GRg1, NR1, Baicalein, Geniposide, Taurine, *β*-asarone could effectively increase proliferation of PC12 cells at 20 μM (*p* < 0.05, *p* < 0.01, Figure 8). GRb1, Geniposide and Taurine showed fine effects with the lowest concentration of 5 μM (*p* < 0.01). Therefore, these compounds except Rhein, Paeoniflorin and Apigenin all increased cell proliferation on *L-*glutamate-induced PC12 cell.

#### Effects of Extract of LP and Its Extracted Fractions on *L*-Glutamate-Induced PC12 Cell Apoptosis

**FIGURE 9:**
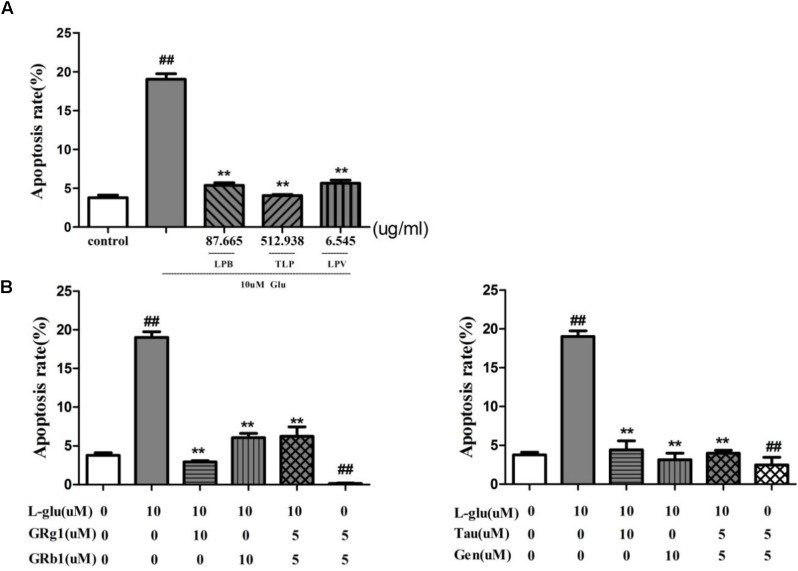
Effect of LP on apoptosis of PC12 cell induced by *L*-glutamate. **(A)** Extract of LP and its extracted fractions. Each group of extract has the highest dose corresponding to the dose of crude medicine at 4000 μg/ml. **(B)** Comounds of LP including Taurine, Geniposide, GRg1, and GRb1. Data are shown as the means ± SEM from three replicate experiments. ^∗^*P* < 0.05, ^∗∗^*P* < 0.01, vs. *L*-Glu; ^#^*P* < 0.05, ^##^*P* < 0.01, vs. control. LPV, volatile oil fraction extract of LP; LPB, *n*-butanol fraction extracts of LP; TLP, total extracts of LP; *L*-Glu, *L*-glutamate; GRg1, Ginsenoside Rg1; GRb1, Ginsenoside Rb1; Tau, Taurine; Gen, Geniposide.

To ascertain the anti-apoptotic effect of LP, apoptosis rate of pretreatment with TLP, LPB, and LPV were evaluated on *L*-glutamate-induced PC12 cell. 10 mM glutamate was able to induce PC12 cells apoptosis from3.79 to 19.03% in 24 h (*P* < 0.01, Figure 9A). However, pretreatment with TLP, LPB, or LPV for 1 h prior to glutamate exposure decreased the apoptosis rate to 5.39% (LPB), 4.06% (TLP), and 5.65% (LPV), respectively (*P* < 0.01). Therefore, LP had obvious neuroprotective effect when PC12 cells were exposed to amino acid damage.

### Effect of Compound Combination in LP

#### Properties Analysis of Total Compounds in LP

For determining whether the six herbal ingredients in LP were different or similar on compound composition, total compounds in six herbs were compared under important properties including MW, nHAcc, nHDon, OB, MlogP, and DL. On one hand, the distribution interval of various properties for all compounds in six medicinal herbs of LP show obvious difference. On the other hand, properties of total compounds of blood-activating herbs such as PS and PN undifferentiated with PR in MW, nHDon, nHAcc, MlogP, and OB, properties of total compounds of heat-clearing herb such as RO only had subtle differences in MW and MlogP compared to RG (*p* < 0.05, Table 3). However, six properties of total compounds in AT for resuscitation and palinesthesia of consciousness almost showed significant differences with other herbs (*P* < 0.01). The results showed that although there were various compounds in the various herbs of LP, the properties of heat-clearing herbs as PR and PS were similar, the same as RO and RG as blood-activating herbs.

**Table 3:** Comparison of compound properties on PR, RO, RG, PS, PN, and AT.

Index	PR	RG	RO	PS	PN	AT
MW	321.68	361.52 (165.53)	442.60 (225.79ˆ##,238.54ˆ*)	350.48 (163.60,172.94)	367.95 (244.27,261.78)	227.44 (140.98ˆ##,149.35ˆ**)
MLogP	2.84	0.06 (4.01ˆ##)	2.44 (3.47,3.82ˆ**)	1.82 (3.48,3.74ˆ**)	3.45 (3.44,3.92ˆ**)	2.99 (2.88,3.25ˆ**)
Hdon	2.42	4.61 (3.27ˆ##)	5.24 (4.08ˆ##,4.28)	3.22 (2.83,3.26ˆ*)	3.02 (4.02,4.47ˆ**)	0.99 (2.55ˆ##,3.22ˆ**)
Hacc	5.14	7.88 (5.43ˆ##)	8.88 (6.35ˆ##,6.40)	6.45 (5.13,5.27)	4.76 (6.43,6.93ˆ**)	2.42 (4.51ˆ##,5.00ˆ**)
OB	32.14	27.28 (23.80)	24.45 (21.79ˆ#,22.53)	31.13 (22.33,23.92)	30.08 (21.01,21.83)	37.97 (20.98ˆ#,22.24ˆ**)
DL	0.3	0.32 (0.27)	0.38 (0.28ˆ#,0.28)	0.37 (0.28,0.28)	0.17 (0.26ˆ##,0.26ˆ**)	0.15 (0.25ˆ##,0.24ˆ**)

*SD, standard deviation; PR, Paeonia lactiflora Pall.; RO, Rheum officinale Baill.; RG, Rehmannia glutinosa (Gaertn.) Li-bosch.; PN, Panax notoginseng (Burk.) F. H. ChenexC. Chow.; PS, Paeonia suffruticosa Andr.; AT, Acorus tatarinowii Schott.; The vital properties include molecular weight (MW); nHAcc, number of acceptor atoms for H-bonds; nHDon, number of donor atoms for H-bonds; DL, drug-likeness; MLogP, Moriguchioctanol–water partition coeff. logP; OB, oralbioavailability. ^∗∗^p < 0.01, ^∗^p < 0.05, vs. RG. ^##^p < 0.01, ^#^p < 0.05, vs. PR*.

#### Effects of Compound Combination in LP on *L*-Glutamate-Induced PC12 Cell Proliferation

According to the prediction result of PI3K/AKT pathway on ICH in LP (Figure 2), effect of compound combination in LP on *L*-glutamate-induced PC12 cell proliferation was further investigated with the three-factor two-level factorial design. The result showed that first-level interactions existed between Taurine and Paeonol, Taurine and Geniposide, Paeonol and Geniposide, Ginsenoside Rg1 and Ginsenoside Rb1 (*p* < 0.01, Table 4), but secondary interaction didn’t exist among compound combination. Taurine was a characteristic compound from BB and Geniposide was an important compound from RG. BB and RG were classic herb pair in TCM, therefore compound combination of taurine and geniposide was reserved for further study. Potential compounds Ginsenoside Rg1 and Ginsenoside Rb1 were from the same herb of PN, which also were reserved to explore the effect of compound combination by PI3K/AKT pathway.

**Table 4:** Results of compound combination in LP on *L*-glutamate-induced PC12 cell proliferation with factorial analysis of three-factor two-level design.

Tset order	Dosage level (μmol/L)			OD450 (*n* = 5)

	Taurine	Paeonol	Geniposide	
1	2.5	2.5	2.5	1.526 ± 0.043
2	2.5	2.5	5.0	1.513 ± 0.087
3	2.5	5.0	2.5	1.502 ± 0.059
4	2.5	5.0	5.0	1.518 ± 0.029
5	5.0	2.5	2.5	1.517 ± 0.078
6	5.0	2.5	5.0	1.314 ± 0.087
7	5.0	5.0	2.5	1.285 ± 0.048
8	5.0	5.0	5.0	1.261 ± 0.087

**Tset order**	**Dosage level(μmol/L)**			**OD450 (*n* = 6)**
	**Ginsenoside Rg1**	**Ginsenoside Rb1**	**Notoginsenoside R1**	

1	2.5	2.5	2.5	1.645 ± 0.053
2	2.5	2.5	5.0	1.626 ± 0.036
3	2.5	5.0	2.5	1.643 ± 0.034
4	2.5	5.0	5.0	1.578 ± 0.053
5	5.0	2.5	2.5	1.533 ± 0.070
6	5.0	2.5	5.0	1.487 ± 0.055
7	5.0	5.0	2.5	1.380 ± 0.061
8	5.0	5.0	5.0	1.283 ± 0.045

*Tests of Between-Subjects Effects: Taurine vs. Paeonol, p < 0.01; Paeonol vs. Geniposide, p < 0.01; Taurine vs. Geniposide, p < 0.01; Ginsenoside Rg1 × Ginsenoside Rb1, p < 0.01. Cells were treated with combined compounds at two kinds of concentration (2.5 and 5 μmol/L) 1 h before and glutamate (10 mM) for additional 24 h. Data are expressed from five replicate experiments as the means ± SEM*.

#### Effects of Compound Combination on *L*-Glutamate-Induced PC12 Cell Apoptosis

Based on the effect of the compound combination of LP in the experiment above (Table 4), combination of Ginsenoside Rg1 and Ginsenoside Rb1, Taurine and Geniposide on neuronal apoptosis were evaluated with the model of *L*-glutamate-induced cell apoptosis. The apoptosis radio of cells was increased from 3.79 to 19.03% after the cells were treated with 10 mM glutamate for 24 h (*p* < 0.01, Figure 9B). The combination of Taurine and Geniposide, Ginsenoside Rg1and Rb1 at 5 μM effectively decreased percentage of apoptosis on *L*-glutamate-induced PC12 cell and normal PC12 cell obviously (*p* < 0.01), and were the same as at 10 μM respectively (*P* < 0.01). The results showed that the combination of Ginsenoside Rg1 and Rb1, taurine and geniposide in LP at low concentration had a significant increase in efficiency on inhibition of neuron apoptosis.

#### Effects of Compounds and Their Combination in LP on PI3K/AKT Pathway

**FIGURE 10:**
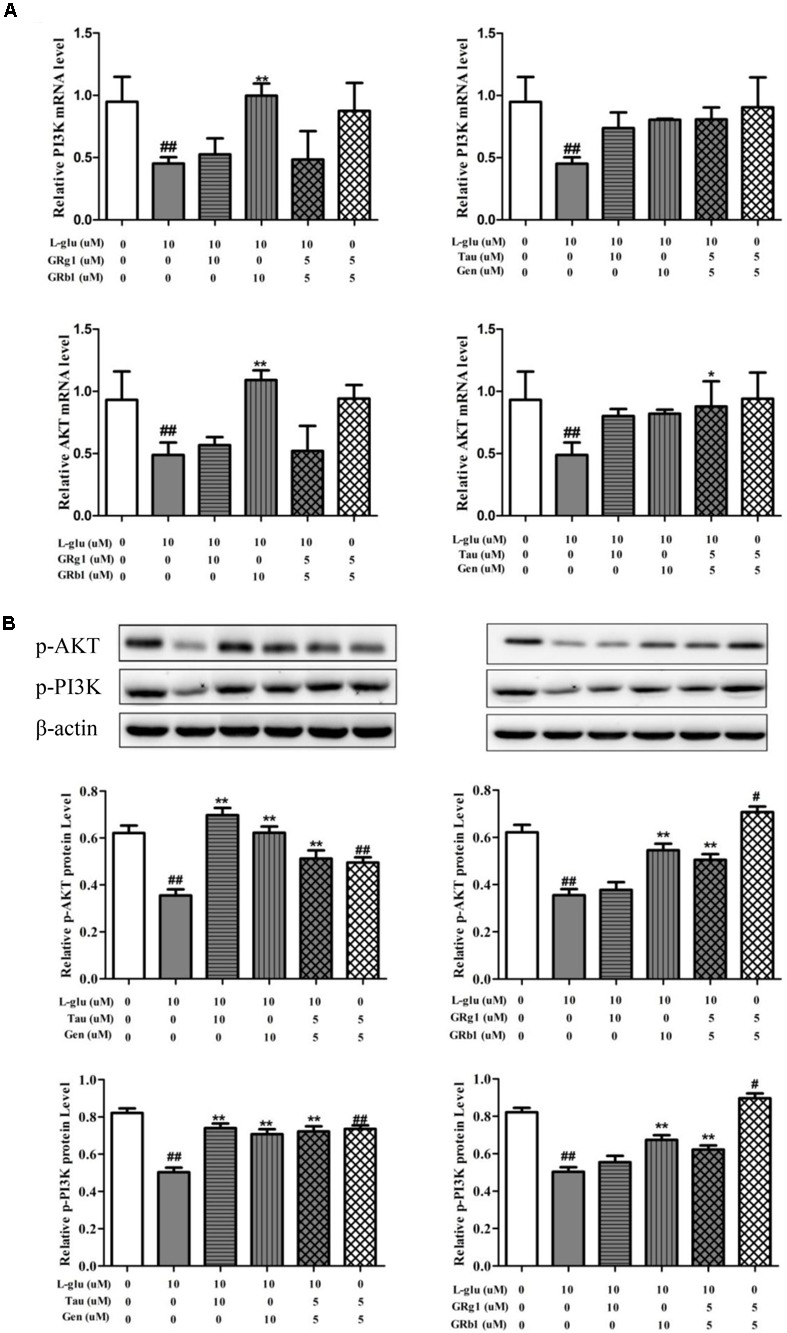
Effects of Taurine, Geniposide, GRg1, and GRb1 in LP on L-Glu-damaged PC12 and normal PC12. **(A)** The PI3K and AKT mRNA level by RT-QPCR. **(B)** The p-AKT and p-PI3K protein level. β-Actin was measured as an internal control. The results are representative of three independent experiments. ^∗^*P* < 0.05, ^∗∗^*P* < 0.01 vs. *L*-Glu; ^#^*P* < 0.05, ^##^*P* < 0.01, vs. control. *L***-**Glu, *L*-glutamate; Tau, Taurine; Gen, Geniposide; GRg1, Ginsenoside Rg1; GRb1, Ginsenoside Rb1.

In order to verify regulation effect of compounds and their combination in LP on PI3K/AKT signaling pathway, the mRNA and protein levels of PI3K and AKT were measured with RT-QPCR and western blot (Figure 10). Compared with normal groups, *L*-Glu-damaged groups showed down-regulated of PI3K and AKT mRNA and protein expression (*P* < 0.01). Compared with *L*-Glu-damaged groups, GRb1 upregulated PI3K and AKT mRNA expression (*P* < 0.01). The combination of taurine and geniposide at 5 μM upregulated AKT mRNA the expression (*P* < 0.05). However, Taurine or Geniposide at 10 μM, and combination of Taurine and Geniposide at 5 μM up-regulated p-AKT and p-PI3K protein expression on *L*-Glu-damaged PC12 cell (*P* < 0.01). GRb1 at 10 μM, and combination of GRb1and GRg1 at 5 μM also up-regulated p-AKT and p-PI3K protein expression on *L*-Glu-damaged PC12 cell (*P* < 0.01). In addition, combination of GRb1and GRg1 at 5 μM up-regulated p-AKT and p-PI3K protein expression on normal PC12, while combination of Taurine and Geniposide at 5 μM down-regulated p-AKT and p-PI3K protein expression on normal PC12. The results showed that Taurine, Geniposide and GRb1 in LP individually up-regulated the level of PI3K and AKT on the level of protein obviously. Furthermore, the combination of Taurine and Geniposide, GRb1 and GRb1 at half-dosage respectively had a significant increase in efficiency on up-regulation of protein expression of PI3K and AKT.

## Discussion

In this study, we have constructed the network pharmacology of LP against ICH based on data-mining and computational modeling. It was performed to investigate biological efficacy of LP for treating ICH *in vivo* and *vitro* experiment. Meanwhile we probed into compound combination in LP on *L*-glutamate-induced PC12 cell. Importantly, our current finding made the complex relation of LP against ICH more unambiguous and provided an insight for understanding the pharmaceutical activity and therapeutic efficacy of LP against ICH.

During the system network construction in mining the relationship between LP and ICH, the P-T-D network containing 34 potential compounds of LP with146 corresponding targets was obtained. Function of targets of compounds in LP covered inflammation, cell apoptosis, oxidative stress, and neurotransmitters. The network integrally displayed LP had a treatment on ICH based on multi-compound interaction with multi-target at the molecular level. In the meanwhile, 142 targets of potential compounds were involved in 24 signaling pathways on ICH, Ligand-dependent caspase activation and intrinsic pathway for Apoptosis occupied a large proportion. ICH has two phases of injury involved. The primary injury emerges from the beginning of bleeding and is primarily due to cerebral vascular injury (Qureshi et al., 2009). Secondary injury might break out with various parallel pathological pathways including excitotoxicity, cytotoxicity of blood, inflammation, blood-brain-barrier disruption, and oxidative stress (Ren et al., 2014). These pathophysiological processes inevitably bring neuronal cell death. PI3K/AKT pathway is a classic pathway which participates in cell survival and proliferation. According to network prediction, we found the PI3K/AKT pathway had close relationship with compounds in LP. Upstream protein PI3K may be regulated by 19 compounds in LP such as Emodin, Baicalein, Ginsenoside Rg1; and downstream protein AKT may be regulated by 17 compounds in LP such as Catalpol, Paeoniflorin, Paeonol, Ginsenoside Rg1.

Hypertension is one of the most common physiological causes of intracerebral hemorrhage. *In vivo* experiment, we found that LP can decrease levels of HS-CRP, TNF-α, NF-kβ and IL-1 on SHR, which implied LP was capable of inhibiting inflammatory responses on SHR. D2D and E2 levels were also decreased after LP administration, which replied LP could activate fibrinolytic reaction and protect blood vessels. Finally, S-100B and NSE level in SHR were decreased remarkably with treatment of LP. When neurons and axons were disrupted, levels of S-100B and NSE are subsequently released in elevated levels, which possess relatively high specificity and sensitivity for neurological damage (Olivecrona et al., 2015). We concluded that LP overall improved hypertension condition based on the activity of anti-inflammatory, anti-coagulation and blood vessel protection. The clonal line PC12 derived from a solid rat adrenal medulla tumor has been used as a dopaminergic neuronal model for *in vitro* studies. *L*-glutamate obviously inhibited cell proliferation and promoted apoptosis. Pretreatment with LP for 1 h prior could obviously inhibit apoptosis and promote proliferation, showing that LP protect neuron from amino acid damage during the process of excitotoxicity.

Multi-component combination was a feature of TCM treatment of diseases. From the properties analysis of ingredients in LP, heat-clearing herb PR was different from blood-activating herb RO. Animal ingredient BB was obviously different from herbal ingredient. However, they were used in combination in LP. Effect of their potential compound combination was preliminarily explored on cell proliferation based on factorial design (Xu et al., 2017). Factorial design is a multi-factor cross-group design, which can not only test the differences between the levels of each factor, but also test the interaction between the factors. The result showed that first-level interactions existed and secondary interaction was not found. First-level interaction existed between Taurine and Paeonol, Taurine and Geniposide, Paeonol and Geniposide. Furthermore, GRg1 and GRb1 derived from PN had the interaction. Meanwhile, we found that Taurine and Geniposide, GRg1 and GRb1 had a significant increase in efficiency at the low concentration on inhibiting the apoptosis of PC12 cell; Ginsenoside Rg1 and Ginsenoside Rb1 at the low concentration had a significant increase in efficiency on the up-regulation expresssion of PI3K and AKT protein. The geniposide and taurine had a s significant increase in efficiency at the low concentration on PI3K and AKT protein when PC12 cell was damaged by glutamate.

This is the first time to study pharmacological effects and therapeutic efficacy of LP on ICH by means of network pharmacology. The research provided new insight for LP against ICH according to the way of “multi-component, multi-target, multi-pathway. LP played a integrated role on cerebral hemorrhage from anti-inflammatory, anti-coagulation, blood vessel protection and protection neuron from excitotoxicity. Meanwhile, compounds in LP such as Taurine, Geniposide, Ginsenoside Rb1 and Ginsenoside Rg1, were verified that they have intervened in PI3K/AKT pathways. Taurine of BB and Paeonol of PS, GRg1 and GRb1 derived from PN had a s significant increase in efficiency on inhibiting the apoptosis of PC12 cells at the low concentration based on PI3K/AKT pathway.

However, network pharmacology research is a perpetual exploration process based on current level of experiments, Further studies should focus on discovering prototype compounds and metabolic compounds in LP based on pharmacodynamics and pharmacokinetic experiments. Interaction of compound includes synergy and antagonism, synergy of multi-component in LP still was needed to systematically study with dose-response in the future. Pathway analysis for targets of potential compounds in LP found a large number of signal pathways was in urgent need of exploring the inner complicated mechanism of LP against ICH.

## Ethics Statement

Experimental animal ethics committee of Nanjing University of Traditional Chinese Medicine approved the experiment.

Application Number: A171101.

Approval Number: ACU 171103.

## Author Contributions

XL and XH developed the model. YT calibrated the model. FZ collected compound information in LP. GL and LY provided suggestions and material support. YC reviewed the manuscript.

## Conflict of Interest Statement

The authors declare that the research was conducted in the absence of any commercial or financial relationships that could be construed as a potential conflict of interest.

## Footnotes

1 http://www.genecards.org

2 http://lsp.nwu.edu.cn/tcmspsearch.php

3 http://www.ncbi.nlm.nih.gov/gene
